# Blockage-Aware Pinching-Antenna-Enabled Mobile Edge Computing for Indoor IoT: Joint Antenna Placement, Serving Order, Task Offloading, and Computing Resource Allocation

**DOI:** 10.3390/s26216725

**Published:** 2026-10-23

**Authors:** Mingming Zhang, Yuhuan Pang, Hui Qin, Yulei Wang

**Affiliations:** Hubei Key Laboratory of Intelligent Wireless Communications, Hubei Engineering Research Center of Intelligent IoT Technology, College of Electronics and Information Engineering (School of Robotics), South-Central Minzu University, Wuhan 430074, China; zhangmingming@mail.scuec.edu.cn (M.Z.); pangyuhuan@mail.scuec.edu.cn (Y.P.); qinhui@mail.scuec.edu.cn (H.Q.)

**Keywords:** pinching-antenna system, mobile edge computing, task offloading, blockage

## Abstract

Indoor millimeter-wave Internet of Things (IoT) terminals are constrained by limited computing capability and energy, while blockage can severely degrade the uplinks required for computation offloading. This paper investigates a pinching-antenna system (PASS)-enabled uplink mobile edge computing (MEC) framework, where a single pinching antenna (PA) is sequentially repositioned along a dielectric waveguide to serve multiple terminals through time-division multiple access (TDMA). Accounting for three-dimensional blockage geometry and PA movement, the PA serving positions, serving order, partial-offloading ratios, and edge computing resources are jointly optimized to minimize a weighted cost of the normalized task latency and the normalized terminal energy consumption under frame-duration, task-deadline, energy-budget, and computing-capability constraints. A two-layer algorithm is developed by combining structured position and order searches with biconvex resource allocation. Simulations show that the proposed design achieves a constraint feasibility rate of 0.96, compared with 0.65 and 0.28 for the fixed- and random-position baselines, and that on jointly feasible realizations it reduces the weighted cost by 13.0% over the multi-resolution grid-search baseline and by 36.5–45.9% over the two position baselines. The results further show that task-oriented PA positioning outperforms rate-oriented placement and that this gain cannot be compensated for by simply increasing the terminal transmit power.

## 1. Introduction

The large-scale deployment of wireless sensor networks and the industrial Internet of Things (IoT) has resulted in visual, vibration and environmental sensing terminals that continuously generate data requiring immediate processing [1]. Tasks such as surface defect inspection, machine-vision sorting and equipment condition diagnosis are not only computation-intensive, but must also close the sensing–computing–decision loop within seconds. These terminals, however, typically have limited processing capability and are powered by capacity-limited batteries, and cannot carry such workloads on their own. Mobile edge computing (MEC) pushes computing and storage capability to the network edge close to the terminals, allowing a terminal to offload all or part of its task to a nearby server and thereby to achieve a better trade-off between task latency and terminal energy consumption [2]. The benefit of MEC is nevertheless constrained by the energy supply of the terminals. Uplink transmission itself consumes precious battery energy, so how much to offload and when to offload must be weighed jointly with energy consumption. Joint optimization of communication and computing resources has consequently attracted extensive research attention. The work in [3] characterized the energy-optimal structure of offloading resource allocation under time-division multiple access (TDMA) and orthogonal frequency-division multiple access (OFDMA), while the study in [4] further jointly optimized the offloaded data volume, the CPU frequency and the time allocation in wireless-powered scenarios. Although these studies demonstrate the potential of MEC systems, their performance remains highly sensitive to the wireless channel conditions: once the channel quality deteriorates, for instance in the presence of obstacle blockage or severe path loss, the efficiency of computation offloading degrades significantly.

Flexible-antenna technologies, such as reconfigurable intelligent surfaces (RISs) [5], movable antennas [6,7] and fluid antennas [8], have recently become a frontier paradigm for actively adjusting wireless channel conditions, and have been introduced into MEC to enhance the offloading links [9,10]. These technologies, however, suffer from inherent limitations. An RIS is subject to the double-fading effect of the cascaded base station–RIS–device link, whereas movable and fluid antennas can typically be repositioned only within a few wavelengths, which is insufficient to combat room-scale path loss and deterministic blockage. These challenges motivate the exploration of an alternative architecture, namely the pinching antenna (PA). First proposed by NTT DOCOMO in 2022 [11], the PA is a novel reconfigurable antenna based on leaky-wave radiation from a slender dielectric waveguide, along which discrete pinching dielectric elements are deployed to locally control signal radiation and reception. By selectively placing these pinching points, a PA can dynamically steer the radiation and establish line-of-sight (LoS) links with nearby terminals [12,13,14]. The key advantage of the PA lies in its physical flexibility. The waveguide can be extended arbitrarily along the ceiling, so that the radiating aperture is brought close to the target terminal and the propagation loss is substantially reduced. Moreover, its hardware implementation requires only the insertion or removal of dielectric elements, which makes it a cost-effective and scalable alternative to conventional reconfigurable antennas. In view of these properties, the PA is particularly suitable for MEC systems, in which the establishment of robust and reconfigurable LoS links is essential.

Research on pinching antennas is still at an early stage, and existing works mainly focus on their potential in wireless communication systems. Downlink rate maximization and uplink minimum-rate maximization for pinching-antenna systems (PASS) were studied in [15] and [16], respectively, and a channel modeling and beamforming optimization framework was established in [17]. Activation strategies for discrete pinching points were investigated in [18], a closed-form characterization of the PASS array gain under fixed antenna spacing was derived in [19], and a performance analysis accounting for in-waveguide attenuation was provided in [20]. As far as blockage effects are concerned, the impact of in-waveguide attenuation on position design under LoS blockage was analyzed in [21]. PASS designs under random line-of-sight/non-line-of-sight (LoS/NLoS) channels and under dynamic channels with blockages were developed in [22,23], and PA positions and beamforming were jointly optimized on the basis of geometric visibility in [24]. Regarding the integration of PASS and MEC, the offloading ratios, the transmit powers and the PA positions were jointly optimized in non-orthogonal multiple access (NOMA)-MEC so as to minimize the maximum task latency [25]. PA deployment in wireless-powered MEC was optimized to maximize the computation capacity [26], and deep reinforcement learning was adopted to jointly design uplink PASS beamforming and task offloading [27].

These works advance PASS along two directions, namely physical-layer design and computation offloading, and in both cases the focus differs from that of the present paper. With respect to the integration with MEC, the existing studies either adopt NOMA access or address wireless power transfer and cross-slot queue evolution. Within the blockage-aware line of work, Ref. [24] is the closest to the present paper, since blockage is likewise treated deterministically rather than through a stochastic LoS probability. The objectives nevertheless differ: physical-layer sum rate is maximized there over several simultaneously radiating PAs, and a blocked user is regarded as unserved, whereas a task-level cost is minimized here under deadline, energy-budget and frame-duration constraints, with an attenuated NLoS component retained. The candidate positions are also obtained differently, in closed form from the blockage geometry here rather than by a surrogate-assisted search combined with a weighted minimum mean-square error–deep deterministic policy gradient (WMMSE-DDPG) scheme. To the best of our knowledge, no existing work jointly optimizes the serving positions, the TDMA serving order, the offloading ratios and the edge computing allocation for a single PA that serves multiple energy-constrained sensing terminals in sequence within one frame. Yet for low-cost indoor sensing networks, this configuration is among the most practical to deploy: it requires only a single ceiling-mounted dielectric waveguide and one movable radiating point, without multiple radio-frequency chains or complex interference management. In this case, the PA serving position simultaneously determines the link quality, the mechanical travel distance and the TDMA waiting time of the subsequent terminals, while the shared edge computing capability makes the offloading decisions of different terminals compete with each other. Typical deployments include smart factories and smart warehouses, where shelves and partitions constitute deterministic blockages. Their positions and dimensions can be obtained from a pre-deployed indoor three-dimensional map or an environment-sensing module, so that environmental awareness can directly serve the joint design of the wireless link and the computing resources.

To fill the above gap, the main contributions of this paper are summarized as follows.

- *First*, we develop a blockage-aware PA-enabled MEC framework. A single movable PA successively reconstructs the LoS link of each terminal through TDMA. Antenna movement, terminal queueing and computing-capability sharing are captured in a unified frame-level model. The serving positions, serving order, partial-offloading ratios and edge computing resources are jointly optimized under frame-duration, deadline, energy-budget and computing-capability constraints.
- *Second*, we develop a two-layer algorithm that exploits the discrete–continuous structure of the joint problem. The outer layer searches over PA positions and serving orders using the blockage geometry and the LoS-interval structure. The inner layer solves the biconvex allocation of offloading ratios and edge computing resources, and a pairwise-swap search avoids factorial enumeration of the serving order. The complexity is polynomial in the number of terminals, and online execution is feasible for up to K=8 under the 10 s frame adopted here.
- *Third*, simulations show that task-oriented PA positioning outperforms rate-oriented placement. Maximizing the instantaneous link rate is therefore not equivalent to minimizing the task-level latency–energy cost. The principal benefit of the considered PA architecture stems from its position adjustability. This gain cannot be compensated for simply by increasing the terminal transmit power.

The remainder of this paper is organized as follows. Section 2 presents the system model and the problem formulation. Section 3 describes the proposed two-layer algorithm. Section 4 specifies the simulation setup and examines the convergence of the algorithm, and Section 5 reports the results and the corresponding discussion. Section 6 concludes the paper and indicates future directions.

## 2. System Model and Problem Formulation

### 2.1. Network Model

**Figure 1:**
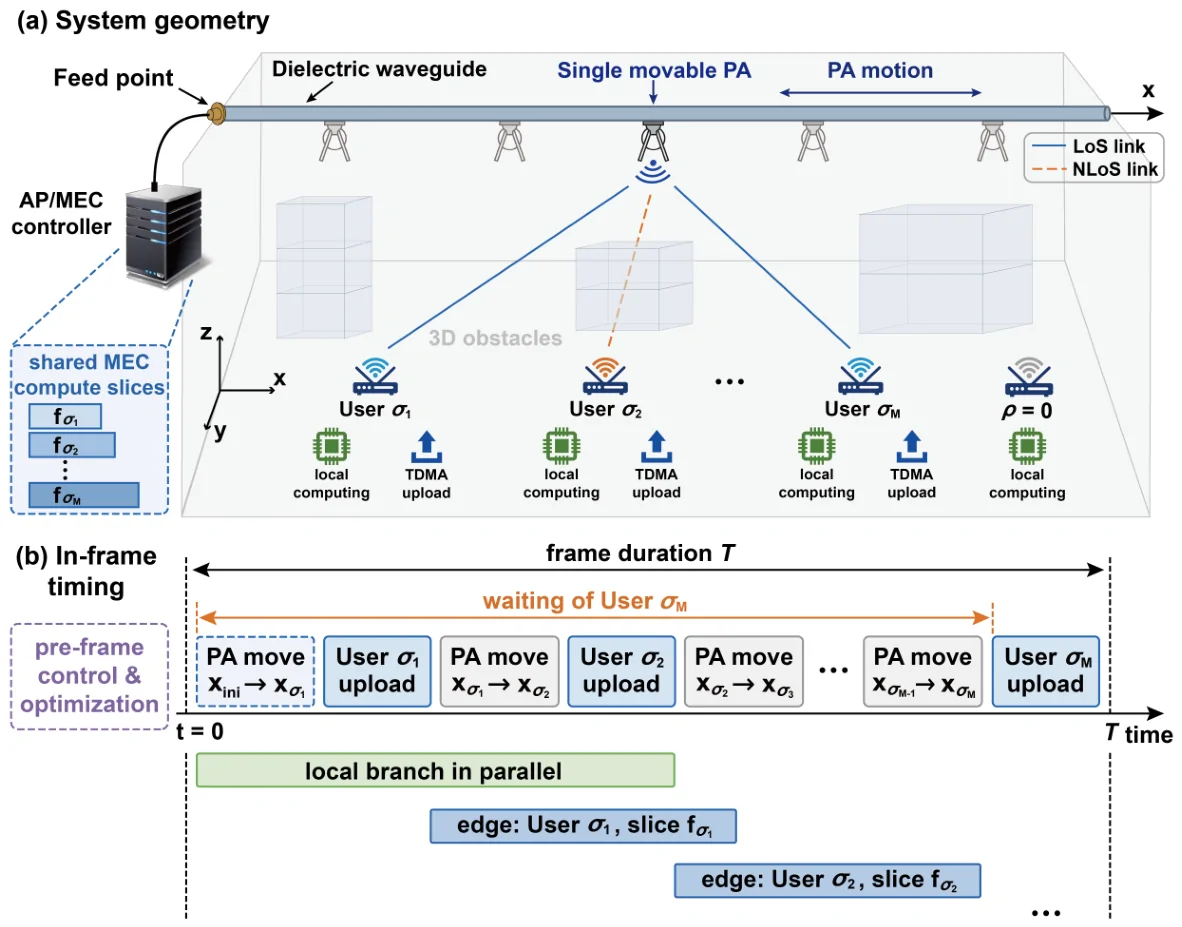
System model: (**a**) geometry of the single pinching-antenna (PA) multi-user edge computing system in a three-dimensional blockage environment; (**b**) in-frame timing of PA movement, time-division multiple access (TDMA) uplink transmission, and parallel local and edge computing.

As illustrated in Figure 1a, we consider a pinching-antenna-assisted uplink MEC system consisting of an access point (AP) integrated with an MEC server and *K* single-antenna terminals, indexed by K={1,…,K}. A dielectric waveguide of length *L* is mounted along the indoor ceiling at a height *H*, with its centerline coinciding with the *x*-axis and its feed point located at $q_{0}={\left[0,0,H\right]}^{T}$. A single PA that can move along the waveguide is deployed on it, and its coordinate when serving user *k* is $q\left(x_{k}\right)={\left[x_{k},0,H\right]}^{T}$ with $0\leq x_{k}\leq L$ [12,25]. Hereafter, $x_{k}$ is referred to as the serving position of user *k*, that is, the antenna placement adopted during its uplink slot, and $x={\left[x_{1},\ldots ,x_{K}\right]}^{T}$ collects the serving positions of the same PA for all terminals across different uplink slots. The PA position is continuously adjusted along the waveguide by a linear actuator, that is, the continuous activation scheme of PASS. This scheme trades the travel latency of the actuator for the highest spatial resolution and is suitable for slowly varying environments such as indoor and industrial IoT [28]. Accordingly, the PA travel time is explicitly included in the in-frame timing [29]. The terminals are fixed at the ground positions $u_{k}={\left[u_{k,x},u_{k,y},0\right]}^{T}$. Each terminal executes its computation-intensive task in the partial-offloading mode: the task is split into two parts, one computed locally and the other offloaded to the MEC server [3].

### 2.2. Blockage Model

Approximating obstacles by regular geometric bodies is a common practice in blockage modeling [24,30]. In this paper, indoor obstacles such as shelves are abstracted as axis-aligned three-dimensional cuboids, whose set is denoted by B; the extent of obstacle b∈B along the three coordinate axes is denoted by $\left[o_{b,a}^{-},o_{b,a}^{+}\right]$ with a∈{x,y,z}. Given a PA position $x_{k}$, the direct link from the PA to user *k* is parameterized as the line segment

(1) $$\ell_{k}\left(s;x_{k}\right)=q\left(x_{k}\right)+s\left[u_{k}-q\left(x_{k}\right)\right],\;0\leq s\leq 1.$$

**Figure 2:**
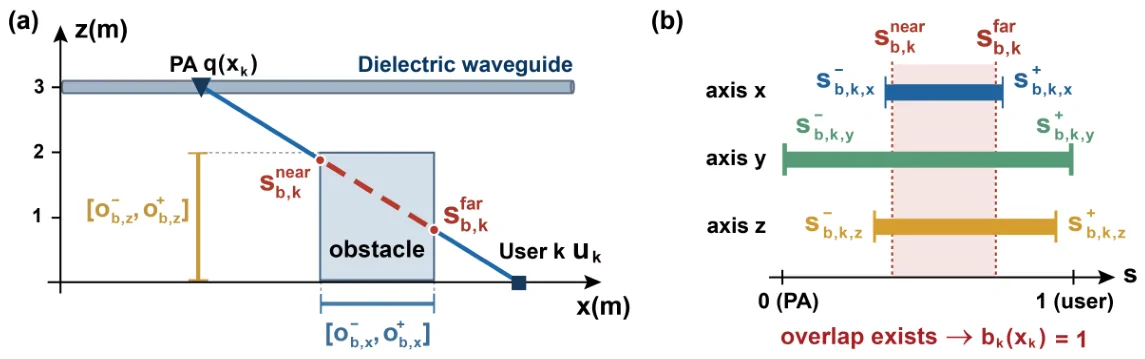
Geometric blockage determination: (**a**) projection of the PA–user link and of one cuboid obstacle onto the *x*–*z* plane; (**b**) interval-overlap test along the three coordinate axes.

The blockage test is illustrated in Figure 2. For each coordinate axis a∈{x,y,z}, the parameter interval $\left[s_{b,k,a}^{-},s_{b,k,a}^{+}\right]$ over which the projection of $\ell_{k}\left(s;x_{k}\right)$ falls within $\left[o_{b,a}^{-},o_{b,a}^{+}\right]$ is first computed. The intersection of the three intervals with [0,1] is

(2) $$\begin{array}{cc} s_{b,k}^{\mathrm{near}} & =\max\left\{0,s_{b,k,x}^{-},s_{b,k,y}^{-},s_{b,k,z}^{-}\right\}, \end{array}$$

(3) $$\begin{array}{cc} s_{b,k}^{\mathrm{far}} & =\min\left\{1,s_{b,k,x}^{+},s_{b,k,y}^{+},s_{b,k,z}^{+}\right\}. \end{array}$$

When this common part is non-empty, that is, when $s_{b,k}^{\mathrm{near}}\leq s_{b,k}^{\mathrm{far}}$, the link penetrates the obstacle. The binary blockage state is therefore given by

(4) $$b_{k}\left(x_{k}\right)=\left\{\begin{array}{cc} 1, & \exists \;b\in B:\;s_{b,k}^{\mathrm{near}}\leq s_{b,k}^{\mathrm{far}},\\ 0, & \mathrm{otherwise}. \end{array}\right.$$

Indoor obstacles such as shelves remain at fixed positions over a time scale of several frames. We therefore assume that the controller has acquired the position and the dimensions of every obstacle in B from a pre-deployed map, a computer-aided design (CAD) model or an environment-sensing module, so that the blockage state of any candidate position can be determined offline from (4) without online probing. The specific sensing means is beyond the scope of this paper, and the present treatment is consistent with the assumption of [24], where PA positions are optimized on the basis of known geometric visibility.

### 2.3. Channel Model

When $b_{k}\left(x_{k}\right)=0$, the link is in the LoS state and the standard channel model of a single-waveguide PASS is adopted [12,17,25]. Denoting the free-space propagation distance by

(5) $$r_{k}\left(x_{k}\right)=\sqrt{{\left(x_{k}-u_{k,x}\right)}^{2}+u_{k,y}^{2}+H^{2}},$$

the LoS complex baseband channel is written as the cascade of a free-space segment and an in-waveguide segment,

(6) $$h_{k}^{\mathrm{LoS}}\left(x_{k}\right)=\frac{\sqrt{\eta }\;e^{-j\frac{2\pi }{\lambda }r_{k}\left(x_{k}\right)}}{r_{k}\left(x_{k}\right)}\cdot e^{-\alpha x_{k}}e^{-j\frac{2\pi }{\lambda_{g}}x_{k}},$$

where $\eta =c^{2}/\left(16\pi^{2}f_{c}^{2}\right)={\left(\lambda /4\pi \right)}^{2}$ is the free-space power gain at a reference distance of one meter, *c* is the speed of light, $f_{c}$ is the carrier frequency and λ is the free-space wavelength [12]. The guided wavelength is $\lambda_{g}=\lambda /n_{\mathrm{eff}}$, where $n_{\mathrm{eff}}$ denotes the effective refractive index [17]. The in-waveguide amplitude attenuation coefficient α is retained here following [21] and is related to the in-waveguide power attenuation $L_{w}$ in dB/m by $\alpha =L_{w}\ln10/20$. Since the phase does not affect the received power in a single-PA single-user transmission, the LoS channel power gain reads

(7) $$G_{k}^{\mathrm{LoS}}\left(x_{k}\right)={\left|h_{k}^{\mathrm{LoS}}\left(x_{k}\right)\right|}^{2}=\frac{\eta }{r_{k}^{2}\left(x_{k}\right)}e^{-2\alpha x_{k}}.$$

When $b_{k}\left(x_{k}\right)=1$, the direct path is obstructed and only the NLoS multipath components contributed by scatterers remain. Following the modeling approach of PASS blockage studies that account for both LoS and NLoS components [22,23], the blocked link is described here by the average power $\mu_{N}^{2}G_{k}^{\mathrm{LoS}}\left(x_{k}\right)$ of the aggregate NLoS component. Here, $\mu_{N}^{2}\ll 1$ is the power ratio of the NLoS scattering to the LoS gain at the same distance. This treatment absorbs the distance dependence of the NLoS component into $G_{k}^{\mathrm{LoS}}$ and avoids introducing an additional NLoS path-loss exponent. It is a first-order treatment: it captures the average power loss caused by blockage, but not the small-scale fluctuation of the aggregate NLoS component. More detailed millimeter-wave fading models, such as the fluctuating two-ray (FTR) model [31], describe that fluctuation explicitly, and computationally efficient evaluations of the FTR model have recently become available [32]. Such a model is not adopted here, because the design variables enter the problem through the average link rate and because $\mu_{N}^{2}$ would remain the dominant unknown in either case. The sensitivity of the results to $\mu_{N}^{2}$ is therefore examined explicitly in Section 5.10. The link power factor is accordingly defined as

(8) $$\chi_{k}\left(x_{k}\right)=\left\{\begin{array}{cc} 1, & b_{k}\left(x_{k}\right)=0,\\ \mu_{N}^{2}, & b_{k}\left(x_{k}\right)=1, \end{array}\right.$$

so that the channel power gain is uniformly written as

(9) $$|h_{k}\left(x_{k}\right){|}^{2}=G_{k}^{\mathrm{LoS}}\left(x_{k}\right)\;\chi_{k}\left(x_{k}\right).$$

Let $p_{k}$ denote the transmit power of user *k*, *B* the bandwidth and $\sigma^{2}=N_{0}BF_{n}$ the receiver noise power, where $N_{0}$ is the noise power spectral density and $F_{n}$ is the noise figure [33]. Under TDMA, each terminal occupies an exclusive time slot and the terminals do not interfere with each other, so the achievable rate is [12,16]

(10) $$R_{k}\left(x_{k}\right)=B\log_{2}\;\left[1+\frac{p_{k}{\left|h_{k}\left(x_{k}\right)\right|}^{2}}{\sigma^{2}}\right].$$

### 2.4. PA Scheduling and Delay Model

A single PA radiates at only one position at a time, so the terminals have to be separated in the time domain. This follows from the single-PA architecture rather than from a general superiority of time division. Environment division multiple access (EDMA), for instance, separates users by placing several PAs at positions matched to the propagation environment, and outperforms PA-enabled time division when several PAs radiate simultaneously [34]; it presupposes, however, several radiating points at the same instant. Serving the terminals one after another allows the PA to be repositioned for each of them in turn, which is precisely the degree of freedom optimized in this paper, and it keeps the uplink free of inter-user interference.

The system operates on frames of duration *T*. Let $D_{k}$ denote the input data size of the task generated by user *k*, and let $\rho_{k}\in \left[0,1\right]$ denote its offloading ratio, so that $\rho_{k}D_{k}$ bits are transmitted to the MEC server while the remaining $\left(1-\rho_{k}\right)D_{k}$ bits are processed locally. Only the terminals with $\rho_{k}>0$ occupy an uplink slot, and they constitute the active set

(11) $$A\left(\rho \right)=\left\{k\in K:\rho_{k}>0\right\},\;M=\left|A(\rho )\right|.$$

The serving order of the active terminals is denoted by a permutation $\sigma =(\sigma_{1},\ldots ,\sigma_{M})$ on A, and the set of all such permutations is denoted by Π(A). The order that sorts the terminals in ascending serving position satisfies

(12) $$x_{\sigma_{1}}\leq x_{\sigma_{2}}\leq \cdots \leq x_{\sigma_{M}}.$$

In this case, the PA travels in a single direction and the total travel distance is $x_{\sigma_{M}}-x_{\sigma_{1}}$, which is the shortest tour of the one-dimensional visiting problem. The PA moves at a speed $v_{a}$, and $x_{\mathrm{ini}}$ denotes its position at the beginning of the frame, so that the travel times to the first terminal and to the subsequent terminals are, respectively,

(13) $$\begin{array}{cc} T_{\sigma_{1}}^{\mathrm{mov}} & =\frac{\left|x_{\sigma_{1}}-x_{\mathrm{ini}}\right|}{v_{a}}, \end{array}$$

(14) $$\begin{array}{cc} T_{\sigma_{i}}^{\mathrm{mov}} & =\frac{\left|x_{\sigma_{i}}-x_{\sigma_{i-1}}\right|}{v_{a}},\;i=2,\ldots ,M. \end{array}$$

According to (10), the uplink transmission time of user *k* is

(15) $$T_{k}^{\mathrm{up}}=\frac{\rho_{k}D_{k}}{R_{k}\left(x_{k}\right)}.$$

The instant at which the upload of user $\sigma_{i}$ starts and the in-frame scheduling time are then given by

(16) $$\begin{array}{cc} S_{\sigma_{i}} & =\sum_{j=1}^{i}T_{\sigma_{j}}^{\mathrm{mov}}+\sum_{j=1}^{i-1}T_{\sigma_{j}}^{\mathrm{up}}, \end{array}$$

(17) $$\begin{array}{cc} T^{\mathrm{sch}} & =\sum_{i=1}^{M}\left(T_{\sigma_{i}}^{\mathrm{mov}}+T_{\sigma_{i}}^{\mathrm{up}}\right). \end{array}$$

When user *k* is served in the *i*th slot, its upload starting instant is written as $S_{k}≜S_{\sigma_{i}}$. The in-frame timing is shown in Figure 1b. The PA movements and the terminal uploads alternate in sequence, and each edge computing slice starts once the corresponding data have arrived. The local branch of every terminal starts at the beginning of the frame and runs independently of that terminal’s place in the serving order, because the residual data $\left(1-\rho_{k}\right)D_{k}$ require no uplink; a terminal has no local branch only in the limiting case $\rho_{k}=1$. Hence, the serving position x, the offloading ratio ρ and the serving order σ are mutually coupled: changing $x_{k}$ affects not only the uplink rate of user *k* but also the PA travel time, while changing $\rho_{k}$ affects both the uplink duration and the set of terminals that need to be visited. Since the control decisions are produced in the pre-frame phase of Figure 1b, the simulations assume that the PA has been repositioned to $x_{\sigma_{1}}$ before the frame starts, so that $x_{\mathrm{ini}}=x_{\sigma_{1}}$ and $T_{\sigma_{1}}^{\mathrm{mov}}=0$; the in-frame travel time then counts only the movements between consecutive terminals.

### 2.5. Computing and Energy Model

Based on the input data size $D_{k}$ defined above, the task of user *k* is further characterized by a computation intensity $\phi_{k}$ in cycles per bit, a deadline $T_{k}^{\max}$ and an energy budget $E_{k}^{\max}$ [1,25]. The number of CPU cycles required to complete the whole task is

(18) $$C_{k}=D_{k}\phi_{k}.$$

Under partial offloading, $\rho_{k}C_{k}$ CPU cycles are executed at the MEC server, while the remaining $\left(1-\rho_{k}\right)C_{k}$ cycles are processed locally. Let $f_{k}^{\mathrm{loc}}$ be the local CPU frequency and $\kappa_{k}$ the effective switched capacitance, so that the CPU power consumption model is $\kappa_{k}{\left(f_{k}^{\mathrm{loc}}\right)}^{3}$ [3]. The local computing latency and energy consumption are then [25]

(19) $$\begin{array}{cc} T_{k}^{\mathrm{loc}} & =\frac{\left(1-\rho_{k}\right)C_{k}}{f_{k}^{\mathrm{loc}}}, \end{array}$$

(20) $$\begin{array}{cc} E_{k}^{\mathrm{loc}} & =\kappa_{k}\left(1-\rho_{k}\right)C_{k}{\left(f_{k}^{\mathrm{loc}}\right)}^{2}. \end{array}$$

The uplink energy consumed for transmitting the offloaded data is [25]

(21) $$E_{k}^{\mathrm{up}}=p_{k}T_{k}^{\mathrm{up}},$$

where $T_{k}^{\mathrm{up}}$ is given in (15). The total computing capability *F* of the edge server, measured in cycles per second, is shared by the offloading terminals. The controller allocates a computing slice $f_{k}\geq 0$ to user *k*; the slices are executed in parallel and satisfy the capacity constraint [9,35]

(22) $$\sum_{k\in A(\rho )}f_{k}\leq F,$$

and the corresponding edge computing latency is

(23) $$T_{k}^{\mathrm{edg}}=\frac{\rho_{k}C_{k}}{f_{k}}.$$

Typical sensing and inference tasks return only labels, alarms or control commands, so that the output data size is far smaller than the input data size, and the transmit power of the AP is much higher than that of the terminals. In line with most MEC studies [1,10,25], the downlink latency of the computation results and the corresponding reception energy of the terminals are therefore neglected. The completion latency of the offloading branch comprises the upload starting instant produced by waiting and movement, the upload latency and the edge computing latency; that is,

(24) $$T_{k}^{\mathrm{off}}=S_{k}+T_{k}^{\mathrm{up}}+T_{k}^{\mathrm{edg}},$$

with $T_{k}^{\mathrm{off}}=0$ when $\rho_{k}=0$. Since the two branches are executed in parallel, the task completion latency is the larger of the two, whereas the total energy consumption at the terminal side consists of local computing and uplink transmission [25]:

(25) $$\begin{array}{cc} T_{k}^{\mathrm{task}} & =\max\left\{T_{k}^{\mathrm{loc}},\;T_{k}^{\mathrm{off}}\right\}, \end{array}$$

(26) $$\begin{array}{cc} E_{k} & =E_{k}^{\mathrm{loc}}+E_{k}^{\mathrm{up}}. \end{array}$$

The energy consumed by the PA actuator and the MEC server is not included in $E_{k}$, since they are assumed to be powered by the network infrastructure rather than by the energy-constrained terminals.

### 2.6. Problem Formulation

The objective is to jointly reduce the task completion latency and the energy consumption of the terminals. Since these two metrics have different physical units and numerical scales, they are normalized with respect to fully local computing. Taking the latency $T_{k}^{\mathrm{loc},0}=C_{k}/f_{k}^{\mathrm{loc}}$ and the energy consumption $E_{k}^{\mathrm{loc},0}=\kappa_{k}C_{k}{\left(f_{k}^{\mathrm{loc}}\right)}^{2}$ as reference quantities, the normalized latency and the normalized energy consumption are defined as

(27) $$\begin{array}{cc} {\bar{T}}_{k} & =\frac{T_{k}^{\mathrm{task}}}{T_{k}^{\mathrm{loc},0}}, \end{array}$$

(28) $$\begin{array}{cc} {\bar{E}}_{k} & =\frac{E_{k}}{E_{k}^{\mathrm{loc},0}}, \end{array}$$

and are combined through a weight ω∈[0,1] into the system cost [36]

(29) $$J\left(x,\rho ,f,\sigma \right)=\sum_{k=1}^{K}\left[\omega {\bar{T}}_{k}+\left(1-\omega \right){\bar{E}}_{k}\right].$$

where $f={\left[f_{1},\ldots ,f_{K}\right]}^{T}$ collects the edge computing slices allocated to all terminals. The waveguide is discretized into a candidate position set X of $N_{x}$ uniformly spaced points for the position search, and the joint optimization problem is formulated as

(30a) $$\begin{array}{cc} P_{1}:\;\underset{x,\rho ,f,\sigma }{\mathrm{minimize}} & J(x,\rho ,f,\sigma ) \end{array}$$

(30b) $$\begin{array}{cccc} \;\mathrm{subject}\;\mathrm{to} & x_{k}\in X,\;0\leq \rho_{k}\leq 1,\;f_{k}\geq 0, & \; & \forall k\in K, \end{array}$$

(30c) $$\begin{array}{cc} \; & \sigma \in \Pi \left(A(\rho )\right), \end{array}$$

(30d) $$\begin{array}{cc} \; & \sum_{k\in A(\rho )}f_{k}\leq F, \end{array}$$

(30e) $$\begin{array}{cccc} \; & T_{k}^{\mathrm{task}}\leq T_{k}^{\max},\;E_{k}\leq E_{k}^{\max},\; & \; & \forall k\in K, \end{array}$$

(30f) $$\begin{array}{cc} \; & T^{\mathrm{sch}}\leq T. \end{array}$$

Constraint (30b) bounds the serving positions and the offloading ratios, and constraint (30c) requires the serving order to be a permutation of the active terminals. Constraint (30d) keeps the computing slices within the total capability of the server, while constraints (30e) and (30f) limit the task completion latency, the terminal energy consumption and the in-frame scheduling time, respectively.

In order to distinguish the degree of violation of infeasible solutions during the search, let ${\left[z\right]}^{+}=\max\left\{z,0\right\}$ denote the positive-part operator and define the normalized violation

(31) $$V=\frac{{\left[T^{\mathrm{sch}}-T\right]}^{+}}{T}+\frac{{\left[\sum_{k\in A(\rho )}f_{k}-F\right]}^{+}}{F}+\sum_{k=1}^{K}\left[\frac{{\left[T_{k}^{\mathrm{task}}-T_{k}^{\max}\right]}^{+}}{T_{k}^{\max}}+\frac{{\left[E_{k}-E_{k}^{\max}\right]}^{+}}{E_{k}^{\max}}\right]$$

together with the penalized evaluation function

(32) Φ(x,ρ,f,σ)=J(x,ρ,f,σ)+μV(x,ρ,f,σ),

where μ>0 is the penalty coefficient. For any feasible solution of $P_{1}$, all constraint violations vanish, so that V=0 and Φ=J.

The non-convexity of $P_{1}$ originates from four sources. The blockage state $b_{k}\left(x_{k}\right)$ switches discretely with the position, and the active set A(ρ) is combinatorial, since forcing a terminal to compute purely locally removes both its travel leg and its time slot from the waiting time of the subsequent terminals. In addition, the serving order σ is a permutation variable whose feasible set varies with the active set, and $\rho_{k}$ and $x_{k}$ are coupled non-convexly in the upload latency.

## 3. Proposed Two-Layer Algorithm

To solve $P_{1}$, we develop a two-layer algorithm that separates the continuous resource allocation from the discrete PA position and serving order design. For given serving positions, active set and serving order, the inner layer jointly optimizes the offloading ratios and the edge computing resources. Based on the resulting allocation, the outer layer updates the PA serving positions and the serving order. The two layers are coordinated until no further improvement in Φ is obtained. The inner layer comprises subproblems (1) and (2) below, and the outer layer comprises subproblems (3) and (4).

Once the serving positions x, the active set A and the serving order σ are fixed, the travel times become constants and both $S_{k}$ and $T^{\mathrm{sch}}$ are affine functions of ρ. Introducing the latency epigraph variables $\tau_{k}$ and the hinge penalty variables $v_{k}$, $w_{k}$ and *u*, the inner resource allocation problem is written as

(33a) $$\begin{array}{cc} \;P_{2}:\;\min_{\rho ,f,\tau ,v,w,u} & \sum_{k\in A}\left[\omega \frac{\tau_{k}}{T_{k}^{\mathrm{loc},0}}+\left(1-\omega \right){\bar{E}}_{k}\left(\rho_{k}\right)\right]+\mu \left(\sum_{k}v_{k}+\sum_{k}w_{k}+u\right) \end{array}$$

(33b) $$\begin{array}{cccc} \;\mathrm{s.t.} & \tau_{k}\geq \frac{\left(1-\rho_{k}\right)C_{k}}{f_{k}^{\mathrm{loc}}},\; & \; & \forall k\in A, \end{array}$$

(33c) $$\begin{array}{cccc} \; & \tau_{k}\geq S_{k}\left(\rho \right)+\frac{\rho_{k}D_{k}}{R_{k}\left(x_{k}\right)}+\frac{\rho_{k}C_{k}}{f_{k}},\; & \; & \forall k\in A, \end{array}$$

(33d) $$\begin{array}{cccc} \; & v_{k}\geq \frac{\tau_{k}-T_{k}^{\max}}{T_{k}^{\max}},\;v_{k}\geq 0,\; & \; & \forall k\in A, \end{array}$$

(33e) $$\begin{array}{cccc} \; & w_{k}\geq \frac{E_{k}\left(\rho_{k}\right)-E_{k}^{\max}}{E_{k}^{\max}},\;w_{k}\geq 0,\; & \; & \forall k\in A, \end{array}$$

(33f) $$\begin{array}{cc} \; & u\geq \frac{T^{\mathrm{sch}}\left(\rho \right)-T}{T},\;u\geq 0, \end{array}$$

(33g) $$\begin{array}{cc} \; & \sum_{k\in A}f_{k}\leq F,\;f_{k}\geq 0,\;0\leq \rho_{k}\leq 1. \end{array}$$

**Proposition 1.** 
*Problem $P_{2}$ is biconvex with respect to the variable blocks (ρ;τ,v,w,u) and f. With f fixed, the subproblem in the remaining variables is a linear program; with **ρ** fixed, the problem in (f,τ,v,w,u) is convex; and the coupling term $\rho_{k}C_{k}/f_{k}$ is quasilinear in $\left(\rho_{k},f_{k}\right)$.*

**Proof of Proposition** **1.** The objective (33a) is linear and all constraints except the coupling term in (33c) are affine. With f fixed, $\rho_{k}C_{k}/f_{k}$ is linear in $\rho_{k}$; with ρ fixed, it is a non-negative multiple of the convex function $1/f_{k}$. Moreover, the sublevel set $\left\{\rho_{k}C_{k}\leq tf_{k}\right\}$ is a half-plane for any t≥0, so that the term is quasilinear [37].    □

(1) *Optimization of the offloading ratios **ρ**.* With $f=f^{\left(t\right)}$ fixed, Proposition 1 implies that all constraints of $P_{2}$ are affine in (ρ,τ,v,w,u), so that the subproblem degenerates into the linear program

(34) $$P_{2a}:\;\min_{\rho ,\tau ,v,w,u}\;\left(33a\right)\;\mathrm{s.t.}\;\left(33b\right)–\left(33g\right),\;f=f^{\left(t\right)},$$

whose global optimum is obtained by an interior-point method. Constraints (33b) and (33c) describe, respectively, the affine decrease of the local branch in $\rho_{k}$ and the affine increase of the offloading branch in $\rho_{k}$, the latter including the slot occupancy of the preceding terminals through $S_{k}\left(\rho \right)$. The competition between the two determines the optimal value of $\rho_{k}$.

(2) *Optimization of the computing resource allocation f.* With $\rho =\rho^{\left(t\right)}$ fixed, the coupling term $\rho_{k}C_{k}/f_{k}$ is a non-negative multiple of the convex function $1/f_{k}$, so that the subproblem is the convex program

(35) $$P_{2b}:\;\min_{f,\tau ,v,w,u}\;\left(33a\right)\;\mathrm{s.t.}\;\left(33b\right)–\left(33g\right),\;\rho =\rho^{\left(t\right)},$$

which is likewise solved to global optimality once ρ is fixed. The dual variable of the total capacity constraint in (33g) gives the competition price of the shared computing capability, and a terminal with a heavier offloading load is granted a larger slice.

The two block subproblems above characterize the structure of $P_{2}$, but alternating between them converges slowly. The inner layer therefore does not alternate. It solves $P_{2}$ jointly by multi-start sequential quadratic programming (SQP), which returns a Karush–Kuhn–Tucker (KKT) point of $P_{2}$ rather than a certified global optimum. Proposition 1 remains useful here, because the constraint-wise quasilinearity makes different starting points converge to a consistent solution in most cases, but it does not by itself certify the global optimality of the returned point. The combinatorial dimension of the active set is handled by *greedy shrinking*. Starting from the full terminal set, $P_{2}$ is solved, and each active terminal is then tentatively removed from the uplink serving sequence and the reduced problem is re-solved. The removal is accepted if the exactly evaluated Φ decreases, and the procedure repeats until no improvement is found, which takes at most *K* rounds.

(3) *Optimization of the PA positions x.* With $\left(\rho^{\left(t\right)},f^{\left(t\right)}\right)$ fixed, the position subproblem remains non-convex because the blockage state $b_{k}\left(x_{k}\right)$ switches discretely with the position, and a per-user one-dimensional update is therefore adopted:

(36) $$x_{k}^{\left(t+1\right)}=\underset{x\in C_{k}}{\arg\min}\;\Phi \left(x,x_{-k}^{\left(t\right)},\rho^{\left(t\right)},f^{\left(t\right)},\sigma^{\left(t\right)}\right),$$

where $x_{-k}$ collects the serving positions of the other terminals and the travel times are updated together with the candidate position. The candidate set $C_{k}$ is constructed explicitly from the blockage geometry: $b_{k}\left(x_{k}\right)$ partitions the waveguide into a finite number of LoS and NLoS intervals, and $G_{k}^{\mathrm{LoS}}\left(x_{k}\right)$ is unimodal within each LoS interval. The set $C_{k}$ therefore collects the rate peak and the endpoints of every interval, and its size is determined by the number of obstacles.

(4) *Optimization of the serving order **σ**.* With the remaining variables fixed, the order subproblem is a permutation optimization problem, whose exhaustive enumeration requires M! evaluations. Here, the ascending-position order in (12) is taken as the initial solution and a pairwise-swap local search is applied: in each round all pairs (i,j) are traversed, $\sigma_{i}$ and $\sigma_{j}$ are swapped and the result is evaluated exactly through (32); a swap is accepted as soon as Φ decreases, and the next round begins, until no further improvement is found. A single round requires at most M(M−1)/2 evaluations, and each evaluation only recomputes the travel times and the queueing instants under the new order, with complexity O(M). The ascending initial solution makes the search start from the order with the shortest tour, while the swap step allows extra travel to be traded for a reduction in queueing latency.

**Overall algorithm and complexity.** Algorithm 1 summarizes the overall procedure: the outer layer updates the serving positions over $\left\{C_{k}\right\}$ according to (36), then performs the order swap step, and finally re-solves the inner problem $P_{2}$. Every candidate solution is screened by the exact scheduling evaluation of Φ and only descending updates are accepted, so that the cost along each search trajectory is monotonically non-increasing and the algorithm terminates at a locally stable solution within a finite number of steps. As for the initialization, three deterministic starting points—the blockage-aware rate peak, the LoS rate peak and the common high-rate position—are used together with $N_{r}$ starting points drawn at random from $\left\{C_{k}\right\}$, which reduces the sensitivity to initialization. Letting $N_{p}=3+N_{r}$ be the number of starting points, $I_{a}$ the upper bound on the outer alternations, $N_{c}$ the number of candidate positions per terminal and Q(K) the cost of one inner solve, the overall complexity is $O\left(N_{p}I_{a}\left[K^{2}N_{c}+K^{3}+K^{2}Q\left(K\right)\right]\right)$, where the $K^{3}$ term stems from the order swap step. The complexity is thus polynomial in the number of terminals, and the factorial enumeration of the serving order is avoided.

**Optimality guarantees.** The guarantees available at different levels are not equally strong. At the level of a single block update, both subproblems are solved to global optimality: $P_{2a}$ is a linear program and $P_{2b}$ is convex once the other block is fixed. At the level of the inner problem, $P_{2}$ is biconvex but not jointly convex, so the multi-start SQP solver returns a KKT point rather than a certified global optimum. Multiple starting points reduce the dependence on initialization, but they do not remove it. Alternating between $P_{2a}$ and $P_{2b}$ would instead make the objective monotonically non-increasing and converge to a partial optimum of the biconvex problem; it is not used here because its convergence is slow.

At the level of the complete algorithm, the outer layer accepts a position or order update only when the exactly evaluated Φ decreases, and both neighborhoods are finite. The procedure therefore terminates after a finite number of steps at a solution that is stable with respect to single-user position moves over $C_{k}$ and to pairwise order swaps. No claim of global optimality for $P_{1}$ is made: the position variables switch the blockage state discretely and the serving order is combinatorial, so $P_{1}$ is non-convex in both. One component of the resulting gap, namely the handling of the serving order, is quantified empirically in Section 5.2, where the proposed algorithm stays within 2.1–2.4% of an exhaustive reference that enumerates every serving order.
**Algorithm 1** Blockage-aware two-layer joint optimization**Require:** 
Terminal parameters, candidate position set X, rate table and blockage table**Ensure:** 
$x^{★},\rho^{★},f^{★},\sigma^{★}$  1:Generate the structured candidate sets $\left\{C_{k}\right\}$, three deterministic position starting points and $N_{r}$ random starting points  2:**for** orderswapstep∈{enabled,disabled} **do**  3:       **for** each position starting point **do**  4:             σ← ascending order of the current positions  5:             **repeat**  6:                   **for** k∈K with $\rho_{k}>0$ **do**  7:                         Subproblem (3): update $x_{k}$ by (36); accept if Φ decreases  8:                   **end for**  9:                   **if** the order swap step is enabled **then**10:                         Subproblem (4): swap pairs in σ; accept if Φ decreases11:                   **end if**12:                   Subproblems (1)–(2): solve $P_{2}$ by multi-start SQP13:                   Greedy shrinking: remove active terminals in turn; accept if Φ decreases14:             **until** Φ no longer decreases or the maximum number of iterations is reached15:             Update the incumbent solution16:       **end for**17:**end for**18:**return** $x^{★},\rho^{★},f^{★},\sigma^{★}$

## 4. Simulation Setup and Convergence Behavior

The simulation parameters are listed in Table 1. The carrier frequency and the noise power spectral density follow the settings commonly adopted in millimeter-wave indoor propagation and PASS-assisted MEC studies [25,33], and the in-waveguide attenuation is taken from [21]. The NLoS attenuation is defined as $A_{N}=-10\log_{10}\mu_{N}^{2}$ and is set to 35 dB by default, which corresponds to a blocked link retaining only scattered components far weaker than the LoS one. This value is consistent with millimeter-wave measurements in indoor office scenarios and with the 3GPP indoor channel model, in which NLoS links are markedly inferior to LoS ones [38,39]. The PA travel speed is set to $v_{a}=5$ m/s, comparable to the traverse speed of industrial linear motor modules. The terminals are randomly distributed over a region of [1,19] m × [−3,3] m. The input data size, the computation intensity and the deadline follow uniform distributions on [10,25] Mbit, [40,100] cycles/bit and [5,10] s, respectively, while the energy budget ranges from 0.75 to 1.10 times the energy consumption of fully local computing. The effective switched capacitance is set to $\kappa_{k}=10^{-28}$, which is the value commonly adopted for the CPU energy model of edge terminals [4]. All terminals share the nominal values of Table 1 in the default setting, whereas terminal-dependent values of $f_{k}^{\mathrm{loc}}$ and $\kappa_{k}$ are examined separately in Section 5.11.

### 4.1. Random Scenario Generation

Obstacles are axis-aligned cuboids. Their number is fixed to |B|=4 in all reported experiments. For each obstacle, the center along the waveguide is drawn uniformly on [4,L−4] m and the corresponding extent on [1.5,2.8] m. The center across the room is drawn uniformly on [−0.5,0.5] m and the corresponding extent on $\left[4,L_{y}\right]$ m, so that an obstacle typically spans most of the room width $L_{y}$. Every obstacle occupies the height interval [0,2.8] m, that is, it rises from the floor to just below the waveguide at H=3 m. The resulting cuboid is finally clipped to the room, so that no obstacle extends beyond $\left[0,L\right]\times \left[-L_{y}/2,L_{y}/2\right]$.

Two rejection steps are omitted from this procedure. Obstacles are drawn independently and are not tested for mutual overlap, so that two of them may intersect and act jointly as a single wider blocker. Terminals are likewise drawn independently of the obstacles and are retained when they fall within the footprint of one, which reproduces the case of a terminal placed immediately behind or beneath a shelf. Both conventions render the environments more adverse, and they apply identically to every scheme.

Within one realization, the terminals and the obstacles are generated once from a single random number generator, seeded from the experiment base seed and the realization index. They are then passed unchanged to all competing schemes, so that the comparison is made on identical geometry and every realization is reproducible. Across realizations and across experiments, the layouts are regenerated independently, each experiment using its own base seed. The scenarios for different numbers of terminals are likewise drawn independently rather than by adding terminals to a common layout. The penalty sensitivity study of Section 5.12 is the sole exception: it reuses the seeds of the main Monte Carlo, so that the effect of μ is isolated from that of the scenario. The showcase scenario of Section 5.3 is likewise a single realization drawn from this distribution with a fixed seed, retained because its blockage pattern is representative.

### 4.2. Benchmark Schemes

The benchmark schemes follow the settings commonly used in the PASS and MEC literature [16,25,27] and are listed below.

1. *Multi-resolution grid search*, which represents the practice of determining PA positions by a per-antenna one-dimensional grid search [25]. It is implemented here as a two-level coarse-to-fine grid: each terminal is first scanned on the coarse grid and then refined within the neighborhood of the best coarse grid point, with the serving order fixed to ascending position.
2. *Rate-optimal PASS*, whose serving positions are fixed at the blockage-aware rate peaks, which isolates the gain of the task-level joint design. The name refers to the per-user rate criterion used to place the antenna and does not imply optimality with respect to the joint problem.
3. *Fixed PA*, which stays at the midpoint of the waveguide and does not move with the terminals.
4. *Random PA*, whose serving positions are drawn uniformly at random along the waveguide.
5. *Conventional fixed-antenna AP*, deployed above the center of the region without a dielectric waveguide.
6. *Full offloading* and *all-local computing*, the two extreme offloading strategies.

For the five position schemes above, the offloading ratios and the computing resource allocation are optimized by the inner solver, so that they differ only in the way the serving positions are determined. The two offloading extremes instead fix the offloading ratio: full offloading sets $\rho_{k}=1$ for every terminal and optimizes only the computing resource allocation at the blockage-aware positions, whereas all-local computing sets $\rho_{k}=0$ and uses no uplink.

### 4.3. Convergence Behavior

**Figure 3:**
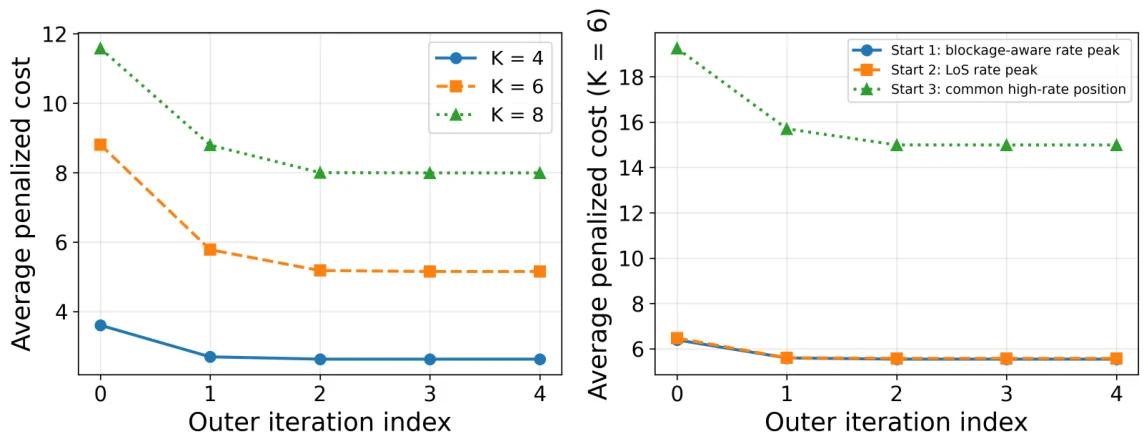
Convergence of the two-layer algorithm: the (**left panel**) shows the average penalized cost of the returned solution against the number of outer iterations for different numbers of terminals, and the (**right panel**) shows the convergence trajectories of the three deterministic starting points for K=6.

The convergence behavior of the algorithm is shown in Figure 3. The left panel plots the average penalized cost against the number of outer iterations, each curve being averaged over 30 random realizations. Since a candidate update is accepted only if it passes the exact scheduling evaluation of Φ, the curves decrease monotonically. For K=4, 6 and 8, the average cost drops from 3.604, 8.809 and 11.585 at the initial solution to 2.626, 5.151 and 7.992, respectively, and convergence is reached within three outer iterations in all cases. The number of iterations does not grow noticeably with the number of terminals, which indicates that $I_{a}=4$ in Table 1 is sufficient. The right panel shows the convergence trajectories of the three deterministic starting points for K=6: the blockage-aware rate peak and the LoS rate peak converge to almost the same cost, namely 5.540 and 5.573, whereas the common high-rate position stalls at a markedly worse local solution of 14.991, about 170.6% higher. The non-convexity of the position subproblem therefore produces local solutions of widely differing quality, so a single initialization is not reliable. In practice, the multi-start strategy relies mainly on the first two starting points.

## 5. Results and Discussion

### 5.1. Evaluation Metrics

The baselines with fixed or random positions fail to satisfy constraints (30d)–(30f) in some random scenarios. In such cases, the penalized cost Φ is dominated by the violation terms, whose magnitude depends on the penalty coefficient μ rather than on the physical overhead, so that using Φ as the only metric would exaggerate the gap between the schemes. The results are therefore reported at three levels. The first is the constraint feasibility rate, that is, the fraction of realizations satisfying all constraints, a realization being counted as feasible when its summed relative constraint violation does not exceed $10^{-9}$. The second is the true weighted cost *J*, computed over the feasible realizations only. The third is the penalized cost Φ, computed over all realizations, which reflects the overall robustness of a scheme in difficult scenarios.

### 5.2. Cost of Simplifying the Serving Order

Serving the terminals in ascending position along the waveguide is the tour with the shortest travel distance and is therefore often adopted directly as a modeling assumption. To assess the cost of this simplification, three schemes are run on 20 random scenarios for each number of terminals. The *ascending order* scheme locks the serving order to the ascending position and optimizes only the serving positions and the resource allocation. The *proposed* algorithm starts from the ascending solution and improves the order by pairwise swaps. The *exhaustive reference* performs a complete position and resource optimization for every order of the active terminals and keeps the best one. All three schemes use the same candidate position set, the same inner solver and the same starting points, so that they differ only in the way the serving order is handled.

**Figure 4:**
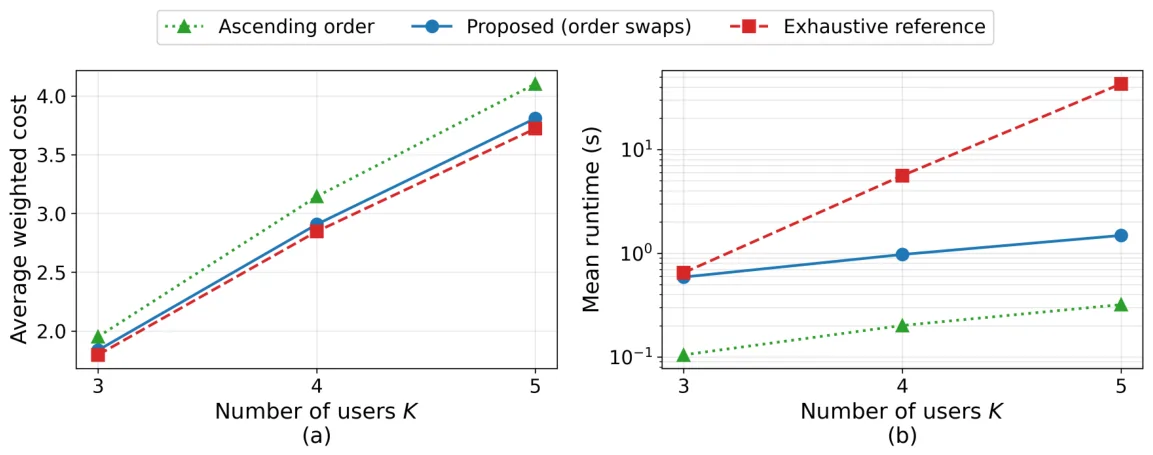
Comparison of three ways of handling the serving order: (**a**) average weighted cost; (**b**) average runtime, with a logarithmic vertical axis.

The results are shown in Figure 4. In terms of cost, the ascending order scheme is the most expensive for every number of terminals, its average cost exceeding that of the exhaustive reference by 8.6% (K=3), 10.5% (K=4) and 10.2% (K=5), whereas the corresponding gaps of the proposed algorithm are 2.2%, 2.1% and 2.4%. The order is therefore worth optimizing: moving a terminal with a tight deadline at the far end of the waveguide earlier in the order saves more queueing latency than the extra travel time it adds. In terms of runtime, the overhead of the exhaustive reference grows with the number of permutations M! and already reaches 42.94 s at K=5, while the proposed algorithm requires only 1.48 s. The pairwise swap thus buys more than an order of magnitude of speed-up at a cost of about 2%, and its polynomial complexity makes it applicable to problem sizes that exhaustive enumeration cannot handle.

### 5.3. Coordination Between Position and Offloading in a Showcase Scenario

In a deterministic scenario with heavy blockage, the serving positions obtained by the joint optimization do not stay at the rate peaks of the individual terminals; instead, they shorten the travel distance between adjacent serving positions while coordinating with the offloading decisions. In this scenario, both the joint design and the rate-optimal PASS satisfy all constraints, and their weighted costs are 2.959 and 3.837, respectively, the former being about 22.9% lower; the fixed PA, the random PA and the conventional fixed AP all violate the constraints. Link rate maximization is therefore not equivalent to task cost minimization.

### 5.4. Comparison with Fixed-Position and Offloading Baselines

Table 2 reports the results of 100 random realizations under the default setting. The joint design outperforms all baselines in terms of both the true cost and the penalized cost. The multi-resolution grid search attains a slightly higher feasibility rate (0.98 against 0.96), yet the Φ and *J* values of the joint design are almost identical, which indicates that its few infeasible realizations involve only mild violations rather than a failure in difficult scenarios. Since *J* is computed for each scheme over its own feasible realizations, it favors the baselines with a low feasibility rate, because their feasible realizations are concentrated in scenarios with light blockage. Repeating the comparison on the 22 realizations in which all six position schemes are simultaneously feasible, the joint design reduces the cost by 13.0%, 21.3%, 36.5%, 29.8% and 45.9% relative to the multi-resolution grid search, the rate-optimal PASS, the fixed PA, the conventional fixed AP and the random PA, respectively. This paired comparison is examined in more detail, together with a significance test, in Section 5.5. The multi-resolution grid search is the strongest baseline. The 13.0% gain of the proposed method over it arises from two sources. The candidate set is determined by the blockage geometry rather than by the grid resolution, which prevents a coarse grid from missing narrow LoS intervals; in addition, the serving order is included in the optimization instead of being fixed to the ascending position. Although full offloading attains a feasibility rate of 0.77, its cost reaches 5.744, which shows that the offloading ratio must be designed continuously. The direct reason for the low feasibility rate of the position baselines is blockage: at the midpoint of the waveguide, 3.44 terminals are blocked on average.

### 5.5. Paired Statistical Comparison

Since all schemes are evaluated on the same random scenarios, a baseline can be compared with the proposed design realization by realization rather than only through averages. This removes the selection bias noted above, by which *J* favors a baseline with a low feasibility rate. Table 3 reports the per-realization difference in true cost between each baseline and the proposed design, restricted to the realizations in which both are feasible. The cost distribution over random blockage layouts is strongly skewed, so significance is assessed by the one-sided Wilcoxon signed-rank test, which does not assume normality, rather than by a *t* test.

Two subsets are reported. In the pairwise subset, each baseline is paired with the proposed design on its own co-feasible realizations, which retains as many pairs as possible, from 27 pairs for the random PA up to 95 for the multi-resolution grid search. In the common subset, only the 22 realizations in which all six position schemes are simultaneously feasible are retained, so that the reductions obtained for different baselines become directly comparable, at the price of a smaller sample and of a bias towards scenarios with light blockage.

Both subsets support the same conclusion. Every paired difference is positive, every 95% confidence interval excludes zero, and every *p* value is below $10^{-4}$. The proposed design is also better in the large majority of the individual realizations, ranging from 77 out of 95 against the multi-resolution grid search in the pairwise subset to 27 out of 27 against the random PA. The smallest margin is the one over the strongest baseline, at 11.1% in the pairwise subset and 13.0% in the common subset, yet it remains highly significant, with $p=9.6\times 10^{-13}$ and $p=6.0\times 10^{-5}$, respectively.

**Figure 5:**
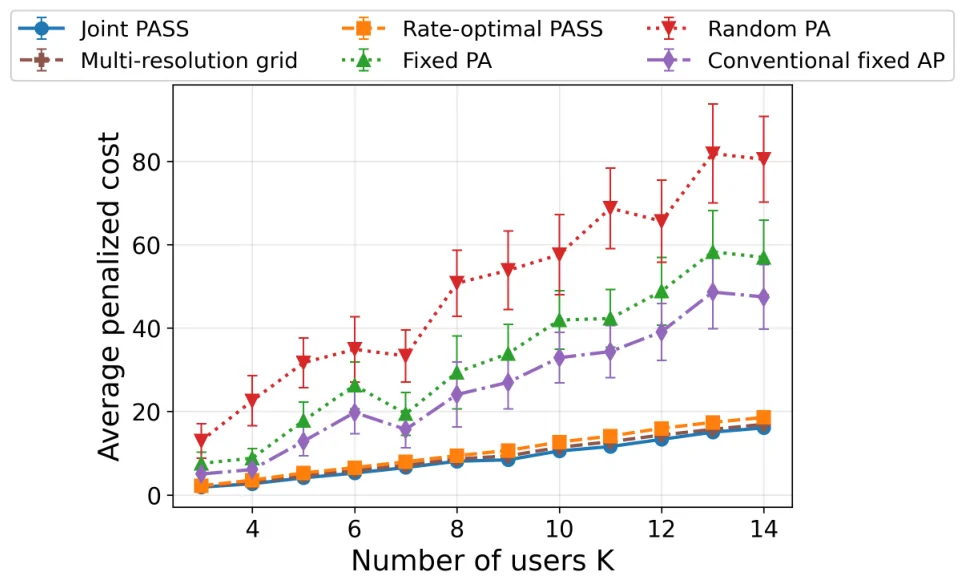
Average penalized cost versus the number of terminals. Error bars denote 95% confidence intervals of the mean over the 30 realizations of each point.

**Figure 6:**
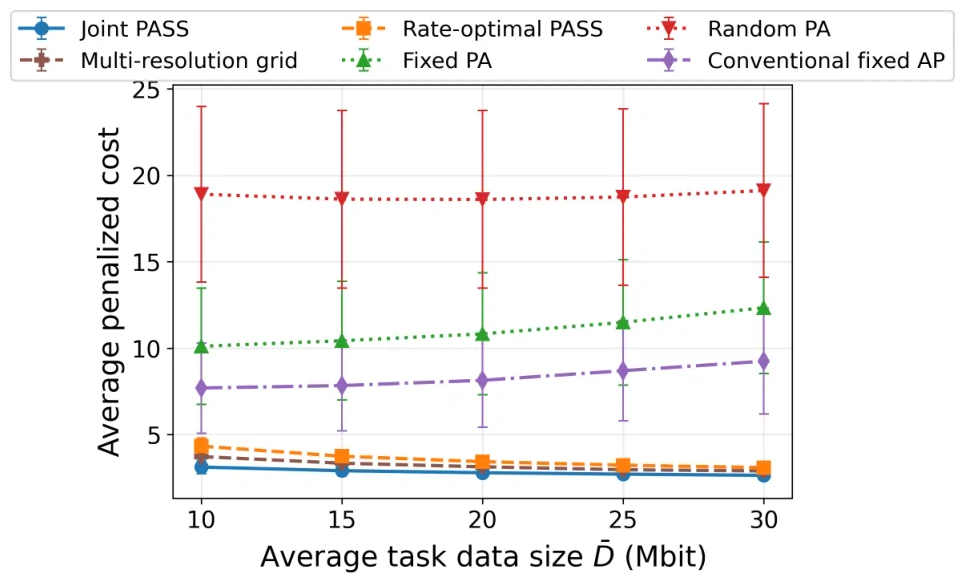
Average penalized cost versus the task data size. Error bars denote 95% confidence intervals of the mean over the 30 realizations of each point.

**Figure 7:**
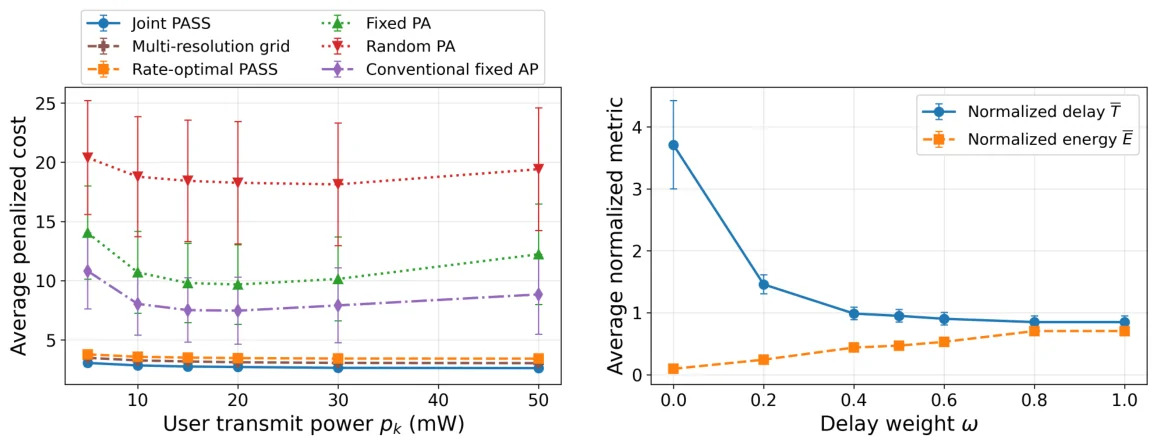
(**Left panel**): average penalized cost versus the terminal transmit power. (**Right panel**): trade-off between the normalized latency and the normalized energy consumption of the joint scheme as a function of the latency weight ω. Error bars in both panels denote 95% confidence intervals of the mean over the 30 realizations of each point.

The same paired comparison was applied at every point of the sweeps of Section 5.6, Section 5.8 and Section 5.9, taken there on the penalized cost of all 30 realizations, which is the quantity plotted in Figure 5, Figure 6 and Figure 7. The error bars of those figures are marginal confidence intervals of each scheme taken separately, and two such intervals may overlap even when one scheme wins on nearly every individual realization. The paired difference is the quantity that settles the comparison. At every sweep point and against every baseline, it is positive with a 95% confidence interval excluding zero. The least favorable case is the multi-resolution grid search at K=13, where the difference is 0.672±0.559 with $p=2.7\times 10^{-2}$; at every other point *p* remains below $2\times 10^{-3}$. The visible overlap of the marginal intervals against the two strongest baselines therefore reflects the spread of the scenario ensemble, not an inconclusive comparison.

### 5.6. Effect of the Number of Terminals

Figure 5 shows the average penalized cost as the number of terminals *K* increases from 3 to 14, each point being averaged over 30 random realizations, with Φ used as the metric. The cost of the joint scheme rises from 1.858 to 16.067, while those of the multi-resolution grid search and the rate-optimal PASS rise to 16.916 and 18.561, the relative gaps remaining stable at 4.5–12.7% and 15.5–32.1%. In contrast, the costs of the fixed PA, the conventional fixed AP and the random PA increase sharply from 7.540, 5.002 and 12.952 to 56.923, 47.437 and 80.420. The more terminals there are, the fiercer the TDMA queueing and the competition for computing capability become, and a fixed or random position cannot accommodate the blockage relations of dispersed terminals. The scenarios for different values of *K* are drawn independently rather than by adding terminals incrementally, so that the baseline curves are not monotone—the fixed PA, for instance, falls back at K=7. All schemes are evaluated on the same scenarios for a given *K*, and these fluctuations are therefore common to all of them. A decreasing feasibility rate with the number of terminals is a common trend: that of the joint scheme falls from 1.00 to 0.70, that of the fixed PA falls from 0.63 to 0, and the random PA has no feasible realization once K≥11. The cost of the fixed PA is 20–51% higher than that of the conventional fixed AP over the whole range, because it is fixed at the midpoint of the waveguide and thus suffers the in-waveguide attenuation without benefiting from any adjustability of the serving position. Under the assumed channel and deployment model, the benefit of PASS is therefore attributable to the adjustability of the serving position: a waveguide whose radiating point cannot be moved does not improve on a conventional fixed AP in the scenarios considered here. The runtime grows polynomially with the number of terminals, amounting to 0.60 s, 4.53 s and 30.24 s for K=3, 8 and 14. The corresponding values of a simplified version with the edge computing capability equally divided are 0.40 s, 2.45 s and 12.02 s, which shows that the overhead stems mainly from the continuous optimization of the resource allocation rather than from the position and order search.

### 5.7. Runtime and Online Feasibility

The positions and offloading decisions returned by the solver are used to configure the next frame, and must therefore be available within one frame. The mean runtime is not a sufficient criterion: it may lie inside the frame even when slower runs overrun it. Online feasibility is therefore judged by the ratio of the 95th-percentile runtime to the frame duration. Table 4 reports the runtime distribution over the same 30 realizations per point, together with the ratios to the frame duration T=10 s.

Two conclusions follow. First, the scheme meets the frame budget at every percentile up to K=8, where the 95th percentile still occupies only 0.64 of a frame. Second, the boundary lies at K=9: the mean runtime is then 0.70 of a frame, but the 95th percentile reaches 1.12 and the worst case 1.28, so a fraction of the frames would miss their deadline. We therefore regard K≤8 as the range in which the algorithm can be executed online frame by frame in its present form, and we do not claim online operation beyond it.

### 5.8. Effect of the Task Data Size

Figure 6 shows the average cost as the average task data size $\bar{D}$, that is, the mean of the per-user data sizes $D_{k}$, increases from 10 Mbit to 30 Mbit, each point being averaged over 30 realizations. The vertical axis is the penalized cost defined in (32), whose latency and energy terms are normalized by the all-local references and therefore reflect a relative overhead. As the task size grows, the absolute latency and energy both increase, but the reference quantities $T_{k}^{\mathrm{loc},0}$ and $E_{k}^{\mathrm{loc},0}$ grow faster, so that the normalized cost decreases—the larger the task, the more pronounced the relative benefit of offloading. The cost of the joint scheme decreases monotonically from 3.12 to 2.64, and the fixed overhead of the PA movement is amortized as well. The multi-resolution grid search decreases from 3.73 to 2.90 and remains above the joint scheme throughout, although the gap narrows from 19.4% to 9.6%, because a larger task size increases the share of the upload time and reduces the relative influence of the position choice. Owing to the linear growth of the upload time and the increasing violation rate, the costs of the fixed PA and the conventional fixed AP rise from 10.11 and 7.69 to 12.34 and 9.24, whereas the random PA is dominated by the blockage penalty and is essentially insensitive to the task size. The advantage of the joint scheme over the fixed-position schemes thus widens as the task load grows.

### 5.9. Transmit Power and the Latency–Energy Trade-Off

The left panel of Figure 7 shows the average cost of each scheme against the terminal transmit power $p_{k}$, each point being averaged over 30 realizations. The joint scheme is not sensitive to the transmit power: as $p_{k}$ increases from 5 mW to 50 mW, its cost decreases only from 3.049 to 2.612. The multi-resolution grid search is equally insensitive, decreasing from 3.481 to 3.031 and remaining about 14% to 16% above the joint scheme over the whole power range. The fixed PA and the conventional fixed AP, in contrast, exhibit a U-shaped behavior. Increasing the power first shortens the upload time, but the upload energy $p_{k}T_{k}^{\mathrm{up}}$ grows linearly with the power and eventually outweighs the logarithmic gain in rate. The two schemes therefore attain their minimum costs of 9.673 and 7.463 around $p_{k}=20$ mW and rise again to 12.229 and 8.836 at 50 mW. The cost of the joint scheme at 5 mW is already lower than that of the fixed-position schemes at their best simulated powers, which shows that the link gain obtained by optimizing the serving position cannot be replaced by increasing the transmit power. The cost of the random PA is dominated by the blockage penalty throughout (above 18), and increasing the power is almost ineffective.

The right panel of Figure 7 shows the average normalized latency and energy consumption of the joint scheme against the weight ω, which characterizes the latency–energy trade-off defined in (29). As ω increases from 0 to 1, the normalized latency decreases from 3.709 to 0.849 and the normalized energy consumption increases from 0.095 to 0.705. For a small ω, the algorithm tends to offload little and to save energy by local computing, whereas for a large ω it offloads aggressively so as to compress the completion latency. For ω≥0.8, the two curves flatten, since the task latency is then determined by the parallel local branch and by the deadline constraint, and further increasing the latency weight no longer changes the offloading decisions. The default value ω=0.5 lies in the trade-off region, with a normalized latency of 0.950 and a normalized energy consumption of 0.469, and thus balances the two metrics.

### 5.10. Sensitivity to the NLoS Attenuation

The NLoS component of a blocked link is represented by the power ratio $\mu_{N}^{2}$, equivalently reported as the attenuation $A_{N}=-10\log_{10}\mu_{N}^{2}$. Whether the conclusions depend on its value is examined by sweeping $A_{N}$ from 0 to 60 dB over 30 realizations. The comparison is against a *LoS-only placement*, which serves each terminal at the position maximizing the unobstructed LoS gain and therefore ignores the blockage indicator, but is charged the true rate at that position. Its remaining variables are optimized as in the proposed design, so the two schemes differ only in the placement rule.

Table 5 reports the results. The proposed design is better over the whole range by between 23.6% and 55.0%, so the qualitative conclusions do not depend on the value of $A_{N}$. Its cost grows monotonically as the NLoS component weakens, from 1.448 at $A_{N}=0$ dB to 3.055 at 40 dB, because progressively fewer positions remain usable for a blocked terminal. Beyond 40 dB, constraint violations begin to dominate and the penalized cost rises to 15.166; a blocked link is already useless at 50 dB, so the values at 50 and 60 dB coincide. The reductions at the two ends have a different origin. Blockage does not affect the channel at $A_{N}=0$ dB, so the 55.0% obtained there reflects the difference between placing the PA by task-level cost and placing it by per-user LoS gain, as in the rate-versus-cost effect of Section 5.4. At the heavily blocked end, the reduction shrinks only because the costs of both schemes grow together and compress their ratio, while the absolute gap first narrows from 1.774 at 0 dB to 1.128 at 35 dB and then widens to 4.681 at 50 dB. The default setting $A_{N}=35$ dB thus lies in the intermediate regime, in which blocked terminals are heavily penalized but not entirely unserviceable, and this is the regime in which optimizing the serving position is most meaningful.

### 5.11. Robustness to Heterogeneous Terminals

Table 1 assigns the same computing capability and the same switched capacitance to every terminal. To test whether the reported gain depends on that choice, $f_{k}^{\mathrm{loc}}$ is drawn uniformly on [0.5,1.5] GHz and $\kappa_{k}$ uniformly on $\left[0.5,1.5\right]\times 10^{-28}$, independently for each terminal, so that the fastest terminal is three times faster than the slowest. The terminal positions, tasks, deadlines and obstacle layouts remain those of the 100 realizations in Table 2; only the terminal parameters change.

Table 6 compares the two settings. The proposed design is barely affected: its feasibility rate moves from 0.96 to 0.95 and its true cost from 2.702 to 2.671. The ranking of the schemes is unchanged, and every paired reduction stays within about two percentage points of its homogeneous value. The fixed-position baselines lose more: the feasibility rate of the fixed PA falls from 0.65 to 0.46 and that of the conventional fixed AP from 0.72 to 0.60. A terminal with a weak processor must offload a larger share of its task and therefore needs a better uplink. A movable PA can be brought close to that terminal during its own slot; a fixed antenna has to serve strong and weak terminals from the same place, and the weak ones are the first to violate their deadline or energy budget. Heterogeneity thus widens rather than narrows the advantage of an adjustable serving position.

### 5.12. Sensitivity to the Penalty Coefficient

The penalty coefficient μ converts constraint violations into cost. It is a parameter of the solver rather than of the system, so the conclusions must be shown not to depend on its value. We sweep μ from 10 to 1000, two orders of magnitude around the default of 100, reusing the seeds of the main Monte Carlo so that the scenarios are identical at every μ.

The penalized cost Φ is not comparable across values of μ, because the same violation is charged μ times: for the fixed PA, whose feasibility rate is only 0.65, the mean Φ grows from 10.90 at μ=100 to 66.02 at μ=1000 purely by this mechanism. Table 7 therefore reports the true cost *J* on the realizations where both schemes are feasible, that is, the paired reduction of Section 5.5.

The ranking of the six placement schemes is identical at every value of μ, with the proposed design first and the random PA last. The paired reductions saturate from μ=100 onwards: the margin over the multi-resolution grid search reads 11.1%, 10.5% and 11.7% at μ=100, 300 and 1000, that over the rate-optimal PASS reads 22.1%, 22.6% and 23.3%, and the feasibility rate of the proposed design reads 0.96, 0.93 and 0.97. The default μ=100 thus lies at the lower edge of a plateau rather than at a tuned optimum.

At μ=10 the penalty is too small, and the solver trades a small deadline or energy overshoot for a lower nominal cost. The feasibility rate of the proposed design therefore falls from 0.96 to 0.78. Every scheme does the same, so the margin over the multi-resolution grid search also narrows to 7.6%. The apparently low true cost of 2.550 is an artifact of the same effect, since it is averaged over the easier 78% of realizations. A coefficient of at least 100 is therefore required, and any larger value gives the same conclusions.

### 5.13. Mechanical Robustness: Positioning Error and Movement Overhead

The model assumes that the actuator places the antenna exactly at the optimized coordinate and that the only mechanical cost is the travel time. Neither assumption holds exactly, so we quantify the effect of relaxing them. The optimized position of every slot is displaced by an independent Gaussian error of standard deviation $\sigma_{p}$, truncated to the waveguide, and the solution is then re-evaluated without re-optimizing. This is the pessimistic open-loop case in which the controller is unaware of the error. Separately, a fixed overhead is added to every repositioning, representing the settling and acknowledgment time that the constant-speed travel model omits.

The movement overhead is benign. As the fixed overhead per repositioning grows from 0 to 0.5 s, the penalized cost rises only from 2.704 to 2.927, an increase of 8.3%, and the feasibility rate falls from 0.96 to 0.95. The reason is that the overhead enters the timeline once per slot and is amortized over an upload lasting seconds, so the frame budget absorbs it.

Positioning error causes two kinds of failure. The first is physical: a displacement can carry the antenna across the edge of an obstacle shadow, turning a LoS link into an NLoS one. The second is numerical: the returned solution usually rests exactly on an active constraint, most often the energy budget, so any displacement produces an overshoot that is nonzero but arbitrarily small, which the zero-tolerance test of Section 5.1 records as a failure. The second kind dominates at small $\sigma_{p}$. At $\sigma_{p}=0.02$ m, 44 of the 47 failures involve no blockage flip, and their median summed relative violation is $1.1\times 10^{-5}$. Table 8 therefore reports the feasibility rate at both the zero tolerance and a relative tolerance of 0.1%, the latter being the physically meaningful measure; the zero tolerance is retained everywhere else in this paper.

At the 0.1% tolerance, the degradation is smooth and the design tolerates centimeter-scale error. An error of $\sigma_{p}=0.02$ m reduces the feasibility rate by 0.04 and raises the true cost from 2.697 to 3.082. Only beyond $\sigma_{p}=0.10$ m does the physical mechanism take over: at least one link flips in 0.27 of the realizations at $\sigma_{p}=0.10$ m and in 0.50 at $\sigma_{p}=0.20$ m, and feasibility falls to 0.81 and 0.72.

## 6. Conclusions

This paper investigated blockage-aware PA-enabled uplink MEC, where a single movable PA sequentially serves multiple energy-constrained terminals through TDMA. A unified frame-level model was developed to jointly characterize three-dimensional blockage, PA repositioning, uplink transmission, partial task offloading and shared edge computing. Based on this model, the PA serving positions, serving order, offloading ratios and edge computing resources were jointly optimized under frame-duration, task-deadline, energy-budget and computing-capability constraints. To solve the resulting mixed discrete–continuous non-convex problem, a two-layer algorithm was developed, combining an outer structured search over PA positions and serving orders with an inner resource allocation that exploits the biconvex structure of the continuous variables.

Three conclusions are drawn from the simulations. First, task-level position design is not equivalent to rate maximization: the cost of the joint scheme is 21.3% lower than that of the rate-optimal PASS. Second, under the assumed channel and deployment model, the dominant benefit of PASS is attributable to the adjustability of the serving position. When the position is not adjustable, the fixed PA attains a feasibility rate of only 0.65 and is even inferior to the conventional fixed AP without a waveguide, whereas the joint scheme reaches a feasibility rate of 0.96 and reduces the true cost of the fixed PA by 36.5% on their co-feasible realizations. Third, this gain cannot be replaced by increasing the transmit power: the cost of the joint scheme at 5 mW is already lower than that of the fixed-position schemes at their best simulated powers. The runtime of the algorithm grows polynomially with the number of terminals. Online frame-by-frame execution is supported up to K=8 under the 10 s frame adopted here, whereas larger instances call for a longer frame or a faster implementation.

The present framework considers a single waveguide with one PA, static terminals and known static obstacle geometry, while PA movement is characterized by a constant-speed travel model with finite positioning resolution. Future work will extend the framework to multiple PAs and multiple waveguides, and thereby to environment division multiple access, investigate coordinated terminal access and PA activation, and incorporate terminal mobility, dynamic blockage and cross-frame task queues. More practical actuator dynamics, including acceleration, settling time, positioning feedback and long-term mechanical constraints, will also be considered, together with validation using ray tracing and measurement-based channel data.

## Figures and Tables

**Table 1:** Simulation parameters.

Parameter	Symbol	Value
Number of terminals	*K*	4
Waveguide length	*L*	20 m
Region width	$L_{y}$	6 m
Waveguide height	*H*	3 m
Carrier frequency	$f_{c}$	28 GHz
System bandwidth	*B*	10 MHz
Terminal transmit power	$p_{k}$	0.01 W
Noise power spectral density	$N_{0}$	−174 dBm/Hz
Receiver noise figure	$F_{n}$	7 dB
In-waveguide power attenuation	$L_{w}$	0.08 dB/m
Local CPU frequency	$f_{k}^{\mathrm{loc}}$	1 GHz
Total edge computing capability	*F*	10 GHz (shared)
Effective switched capacitance	$\kappa_{k}$	$10^{-28}$
PA travel speed	$v_{a}$	5 m/s
Frame duration	*T*	10 s
NLoS attenuation	$A_{N}$	35 dB
Latency weight	ω	0.5
Penalty coefficient	μ	100
Number of candidate positions	$N_{x}$	101
Deterministic position starting points	—	3
Random position starting points	$N_{r}$	5
Inner starting points for ρ	—	3
Maximum outer alternations	$I_{a}$	4

**Table 2:** Performance comparison over 100 random realizations under the default setting. *J* denotes the true weighted cost, computed over the feasible realizations of each scheme; Φ denotes the penalized cost, computed over all realizations; the value after ± is the half-width of the 95% confidence interval.

Scheme	Feasibility Rate	*J* (Feasible)	Φ (All)
Joint PASS (proposed)	0.96	2.702±0.132	2.704±0.128
Multi-resolution grid [25]	0.98	3.054±0.138	3.043±0.137
Rate-optimal PASS	0.93	3.455±0.180	3.491±0.176
Fixed PA	0.65	4.317±0.426	10.904±2.253
Conventional fixed AP	0.72	4.120±0.355	8.462±1.730
Random PA	0.28	4.875±0.657	20.635±3.303
Full offloading	0.77	5.744±0.510	10.831±2.663
All local	0.02	4.000	44.859±3.921

**Table 3:** Paired comparison of the true weighted cost *J* against the proposed joint design, over the 100 realizations of Table 2. The difference is taken as baseline minus proposed on each realization, so that a positive value favors the proposed design; the value after ± is the half-width of the 95% confidence interval. The reduction is the mean difference relative to the mean cost of the baseline itself. The *p* value is that of the one-sided Wilcoxon signed-rank test. Italic row headings identify the pairing subset. The all-local scheme is omitted, because only two realizations are feasible for both it and the proposed design.

Baseline	*n*	Mean Difference	Reduction (%)	Better In	*p*
*Pairwise subset: each baseline paired on its own co-feasible realizations *					
Multi-resolution grid	95	0.337±0.081	11.1	77/95	$9.6\times 10^{-13}$
Rate-optimal PASS	92	0.763±0.144	22.1	90/92	$8.7\times 10^{-17}$
Fixed PA	63	1.714±0.388	39.4	61/63	$5.3\times 10^{-12}$
Conventional fixed AP	70	1.526±0.346	36.8	66/70	$9.0\times 10^{-13}$
Random PA	27	2.199±0.638	44.8	27/27	$7.5\times 10^{-9}$
Full offloading	74	3.183±0.471	55.2	74/74	$3.9\times 10^{-14}$
*Common subset: all six position schemes simultaneously feasible*					
Multi-resolution grid	22	0.394±0.157	13.0	19/22	$6.0\times 10^{-5}$
Rate-optimal PASS	22	0.710±0.351	21.3	21/22	$3.0\times 10^{-5}$
Fixed PA	22	1.511±0.574	36.5	22/22	$2.4\times 10^{-7}$
Conventional fixed AP	22	1.114±0.373	29.8	21/22	$1.2\times 10^{-6}$
Random PA	22	2.229±0.734	45.9	22/22	$2.4\times 10^{-7}$

**Table 4:** Solver runtime against the number of terminals, over 30 realizations per point, with a frame duration of T=10 s. The two ratio columns give the mean and the 95th-percentile runtime relative to *T*, and the last column marks the points whose 95th percentile fits within one frame.

*K*	Mean (s)	95th (s)	Max (s)	Mean/*T*	95th/*T*	Within Frame
3	0.60	0.84	0.90	0.06	0.08	yes
4	0.99	1.29	1.37	0.10	0.13	yes
5	1.61	2.39	2.78	0.16	0.24	yes
6	2.58	3.21	3.52	0.26	0.32	yes
7	3.17	5.07	5.52	0.32	0.51	yes
8	4.53	6.37	8.03	0.45	0.64	yes
9	7.02	11.23	12.79	0.70	1.12	no
10	8.83	13.73	16.42	0.88	1.37	no
11	13.46	20.79	25.29	1.35	2.08	no
12	25.02	40.69	46.60	2.50	4.07	no
13	26.39	52.69	63.32	2.64	5.27	no
14	30.24	53.51	81.77	3.02	5.35	no

**Table 5:** Sensitivity to the NLoS attenuation $A_{N}$ over 30 realizations. The default setting of the paper is 35 dB. Costs are penalized costs Φ.

$A_{N}$ (dB)	Proposed	LoS-Only	Reduction (%)
0	1.448	3.222	55.0
10	1.600	3.287	51.3
20	1.888	3.403	44.5
30	2.309	3.582	35.5
35 (default)	2.612	3.740	30.2
40	3.055	4.486	31.9
50	15.166	19.847	23.6
60	15.166	19.847	23.6

**Table 6:** Effect of terminal heterogeneity, over the same 100 realizations as Table 2. The paired reduction is the reduction in true cost relative to the proposed design on the co-feasible realizations of each baseline, as defined in Section 5.5.

Scheme	Feasibility Rate		Paired Reduction (%)	
	Identical	Diverse	Identical	Diverse
Joint PASS (proposed)	0.96	0.95	—	—
Multi-resolution grid [25]	0.98	0.94	11.1	10.7
Rate-optimal PASS	0.93	0.92	22.1	22.1
Fixed PA	0.65	0.46	39.4	36.8
Conventional fixed AP	0.72	0.60	36.8	38.8
Random PA	0.28	0.26	44.8	46.1
Full offloading	0.77	0.74	55.2	56.1

**Table 7:** Sensitivity to the penalty coefficient μ, over the 100 realizations of Table 2. Feasibility and *J* refer to the proposed design; the remaining columns give the paired reduction in true cost that the proposed design achieves over each baseline on their co-feasible realizations.

	Proposed		Paired Reduction in True Cost (%)				
μ	Feas.	J	Grid	Rate-Opt.	Fixed PA	Fixed AP	Random PA
10	0.78	2.550	7.6	16.8	29.4	28.3	32.5
30	0.90	2.643	9.3	22.3	32.6	32.3	40.1
100	0.96	2.702	11.1	22.1	39.4	36.8	44.8
300	0.93	2.719	10.5	22.6	39.2	36.8	41.4
1000	0.97	2.707	11.7	23.3	38.3	36.9	44.8

**Table 8:** Sensitivity to open-loop positioning error, over the 100 realizations of Table 2. The two feasibility columns differ only in the tolerance applied to the summed relative constraint violation. The flip rate is the fraction of realizations in which the error turns at least one LoS link into an NLoS one, and the true cost is evaluated on the realizations feasible at the 0.1% tolerance.

$\sigma_{p}$ (m)	Mean Shift (m)	Feas. (Zero Tol.)	Feas. (0.1% Tol.)	Flip Rate	*J*
0.00	0.000	0.96	0.97	0.000	2.697
0.02	0.015	0.53	0.93	0.040	3.082
0.05	0.038	0.52	0.90	0.180	3.311
0.10	0.076	0.49	0.81	0.270	3.478
0.20	0.152	0.40	0.72	0.500	4.045

## Data Availability

The original contributions presented in this study are included in the article; further inquiries can be directed to the corresponding author.

## Abbreviations

The following abbreviations are used in this manuscript:

AP	Access point
CAD	Computer-aided design
DDPG	Deep deterministic policy gradient
EDMA	Environment division multiple access
FTR	Fluctuating two-ray
IoT	Internet of Things
KKT	Karush–Kuhn–Tucker
LoS	Line of sight
MEC	Mobile edge computing
NLoS	Non-line of sight
NOMA	Non-orthogonal multiple access
OFDMA	Orthogonal frequency-division multiple access
PA	Pinching antenna
PASS	Pinching-antenna system
RIS	Reconfigurable intelligent surface
SQP	Sequential quadratic programming
TDMA	Time-division multiple access
WMMSE	Weighted minimum mean-square error
